# World Health Organization 2020 guidelines on physical activity and sedentary behaviour

**DOI:** 10.1136/bjsports-2020-102955

**Published:** 2020-11-23

**Authors:** Fiona C Bull, Salih S Al-Ansari, Stuart Biddle, Katja Borodulin, Matthew P Buman, Greet Cardon, Catherine Carty, Jean-Philippe Chaput, Sebastien Chastin, Roger Chou, Paddy C Dempsey, Loretta DiPietro, Ulf Ekelund, Joseph Firth, Christine M Friedenreich, Leandro Garcia, Muthoni Gichu, Russell Jago, Peter T Katzmarzyk, Estelle Lambert, Michael Leitzmann, Karen Milton, Francisco B Ortega, Chathuranga Ranasinghe, Emmanuel Stamatakis, Anne Tiedemann, Richard P Troiano, Hidde P van der Ploeg, Vicky Wari, Juana F Willumsen

**Affiliations:** 1 Physical Activity Unit, Department of Health Promotion, World Health Organization, Geneva, Switzerland; 2 School of Human Sciences, The University of Western Australia, Perth, Western Australia, Australia; 3 Health Promotion Center, Riyadh, Saudi Arabia; 4 Centre for Health Research, University of Southern Queensland, Toowoomba, Queensland, Australia; 5 Age Institute, Helsinki, Finland; 6 Public Health Evaluation and Projection Unit, Finnish Institute for Health and Welfare, Helsinki, Uusimaa, Finland; 7 College of Health Solutions, Arizona State University, Phoenix, Arizona, USA; 8 Department of Movement and Sports Sciences, Ghent University, Belgium; 9 Institute of Technology Tralee, Tralee, Co Kerry, Ireland; 10 UNESCO Chair of Transforming the Lives of People with Disabilities, their Families and Communities, Through Physical Education, Sport, Recreation and Fitness; 11 Healthy Active Living and Obesity Research Group, Children’s Hospital of Eastern Ontario Research Institute, Department of Pediatrics, University of Ottawa, Ottawa, Ontario, Canada; 12 School of Health and Life Sciences, Glasgow Caledonian University, Glasgow, UK; 13 Departments of Medicine, and Medical Informatics & Clinical Epidemiology, Oregon Health & Science University, Portland, Oregon, USA; 14 MRC Epidemiology Unit, School of Clinical Medicine, University of Cambridge, Cambridge, Cambridgeshire, UK; 15 Baker Heart and Diabetes Institute, Melbourne, VIC 3004, Australia; Diabetes Research Centre, University of Leicester, Leicester General Hospital, Leicester, UK; 16 Department of Exercise and Nutrition Science, Milken Institute School of Public Health, The George Washington University, Washington, DC, USA; 17 Department of Sport Medicine, Norwegian School of Sport Science, Oslo, Norway; 18 Department of Chronic Diseases and Ageing, Norwegian Institute of Public Health, Oslo, Norway; 19 NICM Health Research Institute, Western Sydney University, Penrith, New South Wales, Australia; 20 Division of Psychology and Mental Health, University of Manchester, Manchester, UK; 21 Department of Cancer Epidemiology and Prevention Research, CancerControl Alberta, Alberta Health Services, Calgary, Alberta, Canada; 22 Centre for Public Health, Queen's University Belfast, Belfast, UK; 23 Department of Non-Commuicable Diseases, Ministry of Health, Nairobi, Kenya; 24 Centre for Exercise, Nutrition & Health Science, School for Policy Studies, University of Bristol, Bristol, UK; 25 Population and Public Health Sciences, Pennington Biomedical Research Center, Baton Rouge, Louisiana, USA; 26 Research Centre for Health through Physical Activity, Lifestyle and Sport, Division of Exercise Science and Sports Medicine, Department of Human Biology, Faculty of Health Sciences, University of Cape Town, Cape Town, South Africa; 27 Department ofEpidemiology and Preventive Medicine, University of Regensburg, Germany; 28 Faculty of Medicine and Health Sciences, Norwich Medical School, University of East Anglia Faculty of Medicine and Health Sciences, UK; 29 PROFITH (PROmoting FITness and Health through physical activity) research group, Department of Physical Education and Sports, Faculty of Sport Sciences, Research Institute of Sport and Health, University of Granada, Spain; 30 Sports and Exercise Medicine Unit and Department of Allied Health Sciences, Faculty of Medicine, University of Colombo, Colombo, Sri Lanka; 31 Charles Perkins Centre, University of Sydney, School of Health Sciences, Faculty of Medicine and Health, The University of Sydney, Sydney, New South Wales, Australia; 32 Institute for Musculoskeletal Health, The University of Sydney, Sydney, New South Wales, Australia; 33 Epidemiology and Genomics Research Program, National Cancer Institute, Bethesda, Maryland, USA; 34 Department of Public and Occupational Health, Amsterdam Public Health Research Institute, Amsterdam Vrije Universiteit, Amsterdam, The Netherlands; 35 Prevention Research Collaboration, School of Public Health, The University of Sydney, Sydney, New South Wales, Australia; 36 National Department of Health, Port Moresby, Papua New Guinea

**Keywords:** prevention, health promotion, non-communicable disease, physical activity, public health

## Abstract

**Objectives:**

To describe new WHO 2020 guidelines on physical activity and sedentary behaviour.

**Methods:**

The guidelines were developed in accordance with WHO protocols. An expert Guideline Development Group reviewed evidence to assess associations between physical activity and sedentary behaviour for an agreed set of health outcomes and population groups. The assessment used and systematically updated recent relevant systematic reviews; new primary reviews addressed additional health outcomes or subpopulations.

**Results:**

The new guidelines address children, adolescents, adults, older adults and include new specific recommendations for pregnant and postpartum women and people living with chronic conditions or disability. All adults should undertake 150–300 min of moderate-intensity, or 75–150 min of vigorous-intensity physical activity, or some equivalent combination of moderate-intensity and vigorous-intensity aerobic physical activity, per week. Among children and adolescents, an average of 60 min/day of moderate-to-vigorous intensity aerobic physical activity across the week provides health benefits. The guidelines recommend regular muscle-strengthening activity for all age groups. Additionally, reducing sedentary behaviours is recommended across all age groups and abilities, although evidence was insufficient to quantify a sedentary behaviour threshold.

**Conclusion:**

These 2020 WHO guidelines update previous WHO recommendations released in 2010. They reaffirm messages that some physical activity is better than none, that more physical activity is better for optimal health outcomes and provide a new recommendation on reducing sedentary behaviours. These guidelines highlight the importance of regularly undertaking both aerobic and muscle strengthening activities and for the first time, there are specific recommendations for specific populations including for pregnant and postpartum women and people living with chronic conditions or disability. These guidelines should be used to inform national health policies aligned with the *WHO Global Action Plan on Physical Activity 2018–2030* and to strengthen surveillance systems that track progress towards national and global targets.

## Introduction

In 2018, the World Health Assembly (WHA) approved a new *Global Action Plan on Physical Activity (GAPPA) 2018–2030* 1 and adopted a new voluntary global target to reduce global levels of physical inactivity in adults and adolescents by 15% by 2030. As part of the WHA Resolution (WHA71.6), Member States requested that WHO update the 2010 Global Recommendations on Physical Activity for Health.^2^

Global and national guidelines on physical activity are a central component of a comprehensive and coherent governance and policy framework for public health action. WHO recommends all countries establish national guidelines and set physical activity targets. To help support populations to achieve the targets and maintain healthy levels of physical activity, all countries are advised to develop and implement appropriate national and subnational policies and programmes to enable people of all ages and abilities to be physically active and improve health.

Given that the most recent global estimates show that one in four (27.5%) adults^3^ and more than three-quarters (81%) of adolescents^4^ do not meet the recommendations for aerobic exercise, as outlined in the 2010 *Global Recommendations on Physical Activity for Health*,^2^ there is an urgent need to increase priority and investment directed towards services to promote physical activity both within health and other key sectors. These data also reveal no overall improvement in global levels of participation over the last two decades and substantial gender differences.^3 4^ Furthermore, national data consistently show inequalities in participation by age, gender, disability, pregnancy, socioeconomic status and geography,^1^ amplifying the need to intensify investment in physical activity.

This paper reports on the development of new WHO guidelines on physical activity and sedentary behaviour.^5^ These guidelines provide evidence-based public health recommendations concerning the amount (frequency, intensity, duration) and types of physical activity that offer significant health benefits and mitigate health risks (for definitions see table 1). These guidelines have been developed for children, adolescents, adults, older adults and, for the first time, include specific recommendations on physical activity for pregnant and postpartum women and people living with chronic conditions or disability. In addition, for the first time, these WHO guidelines address the health impact of sedentary behaviour. The new WHO guidelines update previous WHO recommendations on physical activity for health released in 2010^2^ with the most recent advances in the evidence base for these behaviours and associated selected health consequences. These new guidelines, together with the *Guidelines on Physical Activity, Sedentary Behaviour and Sleep for Children Under 5 Years of Age*,^6^ provide evidence-updated recommendations for physical activity and sedentary behaviour across the life course.

**Table 1:** Glossary of terms

Term	Definition
Aerobic physical activity	Activity in which the body’s large muscles move in a rhythmic manner for a sustained period of time. Aerobic activity—also called endurance activity—improves cardiorespiratory fitness. Examples include walking, running, swimming and bicycling.
Balance training	Static and dynamic exercises that are designed to improve an individual’s ability to withstand challenges from postural sway or destabilising stimuli caused by self-motion, the environment or other objects.
Bone-strengthening activity	Physical activity primarily designed to increase the strength of specific sites in bones that make up the skeletal system. Bone-strengthening activities produce an impact or tension force on the bones that promotes bone growth and strength. Examples include any type of jumps, running and lifting weights.
Disability	From the International Classification of Functioning, Disability and Health, an umbrella term for impairments, activity limitations and participation restrictions, denoting the negative aspects of the interaction between an individual (with a health condition) and that individual’s contextual factors (environmental and personal factors).
Domains of physical activity	Physical activities can be undertaken in various domains, including one of more of the following: leisure, occupation, education, home and/or transport.
Household domain physical activity	Physical activity undertaken in the home for domestic duties (such as cleaning, caring for children, gardening, etc).
Leisure-domain physical activity	Physical activity performed by an individual that is not required as an essential activity of daily living and is performed at the discretion of the individual. Examples include sports participation, exercise conditioning or training and recreational activities such as going for a walk, dancing and gardening.
Light-intensity physical activity (LPA)	On an absolute scale, light intensity refers to physical activity that is performed between 1.5 and 3 METs. On a scale relative to an individual’s personal capacity, light-intensity physical activity is usually a 2–4 on a rating scale of perceived exertion scale of 0–10. Examples include slow walking, bathing or other incidental activities that do not result in a substantial increase in heart rate or breathing rate.
Metabolic equivalent of task (MET)	The metabolic equivalent of task, or simply metabolic equivalent, is a physiological measure expressing the intensity of physical activities. One MET is the energy equivalent expended by an individual while seated at rest, usually expressed as mLO^2^/kg/min.
Moderate- intensity physical activity (MPA)	On an absolute scale, moderate-intensity refers to the physical activity that is performed between 3 and <6 times the intensity of rest (METs). On a scale relative to an individual’s personal capacity, MPA is usually a 5 or 6 on a rating scale of perceived exertion scale of 0–10.
Moderate-to-vigorous intensity physical activity (MVPA)	On an absolute scale, MVPA refers to the physical activity that is performed at >3 METs (ie, >3 times the intensity of rest). On a scale relative to an individual’s personal capacity, MPA is usually a 5 or above on a scale of 0–10.
Multicomponent physical activity	Multicomponent physical activity are activities that can be done at home or in a structured group or class setting and combine all types of exercise (aerobic, muscle strengthening and balance training) into a session, and this has been shown to be effective. An example of a multicomponent physical activity programme could include walking (aerobic activity), lifting weights (muscle strengthening) and could incorporate balance training. Examples of balance training can include walking backwards or sideways or standing on one foot while doing an upper body muscle-strengthening activity, such as bicep curls. Dancing also combines aerobic and balance components.
Occupation domain physical activity	See work domain physical activity.
Physical activity (PA)	Any bodily movement produced by skeletal muscles that requires energy expenditure.
Physical inactivity	An insufficient physical activity level to meet present physical activity recommendations.
Recreational screen time	Time spent watching screens (television (TV), computer, mobile devices) for purposes other than those related to school or work.
Sedentary screen time	Time spent watching screen-based entertainment while sedentary, either sitting, reclining or lying. Does not include active screen-based games where physical activity or movement is required.
Sedentary behaviour	Any waking behaviour characterised by an energy expenditure of 1.5 METs or lower while sitting, reclining or lying. Most desk-based office work, driving a car and watching television are examples of sedentary behaviours; these can also apply to those unable to stand, such as wheelchair users. The guidelines operationalise the definition of sedentary behaviour to include self-reported low movement sitting (leisure time, occupational and total), TV viewing or screen time and low levels of movement measured by devices that assess movement or posture.
Transport domain physical activity	Physical activity performed for the purpose of getting to and from places, and refers to walking, cycling and wheeling (ie, the use of non-motorised means of locomotion with wheels, such as scooters, roller-blades, manual wheelchair, etc). In some contexts, operation of a boat for transport could also be considered transport-related physical activity.
Vigorous-intensity physical activity (VPA)	On an absolute scale, vigorous intensity refers to physical activity that is performed at 6.0 or more METs. On a scale relative to an individual’s personal capacity, VPA is usually a 7 or 8 on a rating scale of perceived exertion scale of 0–10.
Work domain physical activity	Physical activity undertaken during paid or voluntary work.

The primary audiences and users of these guidelines are policy makers in ministries of health, education, sport, transport, environment, social or family welfare and related sectors, working in high-income as well as low-income and middle-income countries (LMICs), who formulate country-specific guidelines and who develop national or subnational plans and programmes to increase physical activity and reduce sedentary behaviours across the life course. Additional key users of these guidelines include researchers and those working in health services providing advice and guidance (such as community health workers, primary, secondary or tertiary nurses or doctors), allied health and exercise professionals and non-governmental organisations. Communication of these guidelines to members of the public is essential and requires tailoring of the core messages to appropriate and accessible language and formats relevant to cultural contexts in order to be effective.

## Methods and process for developing the WHO guidelines

The guidelines were developed in accordance with the processes set out in the WHO Handbook for Guidelines Development^7^ and commenced in 2019. A Guideline Development Group (GDG) was established comprising relevant experts from required disciplines as well as policy makers and end users of the recommendations, with regional and gender balance. Details of the members of the GDG are available.^5^

At the first meeting in July 2019, the GDG reviewed and finalised the scope of the guidelines and agreed on the set of population, intervention or exposure, comparator and outcome (PI/ECO) questions and critical and important outcomes to be assessed (table 2). The GDG did not include sleep as a behaviour within the scope of these guidelines but did recognise sleep as an important health outcome when considering the impact of physical activity. Between August and February 2020, the WHO secretariat coordinated the commissioning of literature searches and systematic evidence reviews and the GDG subworking groups met virtually to review, summarise and draft preliminary recommendations.

**Table 2:** Criteria for determination of the certainty of evidence (A) and interpretation of the strength of recommendations (B)

**A**	**Criteria for determining the certainty of evidence**
High	Very confident that the true effect lies close to that of the estimate of the effect.
Moderate	Moderately confident in the effect estimate. The true effect is likely to be close to the estimate of the effect, but there is a possibility that it is substantially different.
Low	Confidence in the effect estimate is limited. The true effect may be substantially different from the estimate of the effect.
Very low	Very little confidence in the effect estimate. The true effect is likely to be substantially different from the estimate of effect.
**B**	**Interpretation of the strength of recommendation**
Strong recommendation	Strong recommendations communicate the message that the guideline is based on the confidence that the desirable effects of adherence to the recommendation outweigh the undesirable consequences.
Conditional recommendations	Conditional recommendations are made when there is less certainty about the balance between the benefits and harms or disadvantages of implementing a recommendation, or if the recommendations might not be applicable to all the population group.

### Updating searches and new evidence reviews

The WHO guidelines on physical activity and sedentary behaviour were developed by using, and systematically updating, the evidence collated for the development of other recent national physical activity guidelines that met the following three criteria: (1) the evidence reviews had been conducted according to standard and rigorous systematic processes that were well documented; (2) the assessment of the certainty of the evidence used the Grading of Recommendations Assessment, Development and Evaluation (GRADE) method or an equivalent methodology that was clearly described and documented and (3) the evidence reviews addressed the populations of interest with no restrictions to country or country income level.

For these guidelines on children and adolescents, systematic reviews undertaken by Poitras *et al*,^8^ Carson *et al* 9 and Okely *et al* 10 were used and updated. For pregnant women, the systematic review conducted to inform the 2019 Canadian Guideline for Physical Activity Throughout Pregnancy^11^ was used and updated. For all other age and subpopulation groups, the scientific report^12^ of the Physical Activity Guidelines Advisory Group developed to inform the Physical Activity Guidelines for Americans, second edition^13^ was used and updated. Where gaps in existing evidence were identified, new umbrella reviews were commissioned and full details of these are available elsewhere.^5^

To update the systematic reviews, an agreed set of search terms, databases and search methods, as well as standardised data extraction protocols, were employed to update the evidence. A search for systematic reviews and pooled analyses of cohort studies was conducted for the period from 2017 up to September 2019. The following databases were searched: PubMed, CINAHL, MEDLINE, EMBASE, PsychInfo, SportDiscus and Cochrane to identify reviews that were peer-reviewed, written in English with no restriction on country or country income group and inclusive of reviews including studies using subjective or objectively measured physical activity or sedentary behaviour. Searches were limited to the English language, due to resource constraint and previous experience in the field indicating that other language searches yielded very few, if any additional reviews.^14^ Table 3 provides a summary of the health outcomes assessed for each subpopulation.

**Table 3:** Summary of health outcomes (in alphabetical order) by population groups addressed in the 2020 global guidelines evidence reviews

	5–17 years PA and SB	Adults 18–64 years PA	Adults over 18 years SB	Adults over 65 years PA	Pregnancy and post partum	Chronic conditions*	Children and adults with disability*
**Outcomes**	**Importance†**						
Adiposity—weight gain, weight change, weight control, weight stability, weight status and weight maintenance	Critical	Critical	Critical	Critical	Critical		
Adverse events	Critical	Critical		Critical	Critical		
All-cause and cause-specific mortality		Critical	Critical	Critical		Critical—cancer and cancer-specific	
Bone health	Critical						
Cardiometabolic health	Critical						
Cognitive outcomes	Critical	Critical		Critical			Critical—MS, PD, Stk, Sch, ADHD
Delivery complications					Important		
Disease progression						Critical—HT, T2D, HIV Critical—cancer recurrence	
Falls and fall-related injuries				Critical			
Fetal outcomes					Critical		
Functional ability				Critical			
Gestational diabetes mellitus					Critical		
Gestational hypertension/pre-eclampsia					Critical		
Health-related quality of life		Important		Important		Critical—HT, T2D, HIV	Critical—MS, SCI, ID, MCD, Sch
Incidence of cancer		Critical	Critical	Critical			
Incidence of CVD		Critical	Critical	Critical			
Incidence of hypertension		Important		Important			
Incidence of type 2 diabetes		Critical	Critical	Critical			
Mental health (symptoms of anxiety and depression)	Critical	Critical		Critical	Critical		
Osteoporosis				Critical			
Physical fitness	Critical						
Physical function						Critical—HT, T2D, HIV	Critical—MS, SCI, ID, PD, Stk
Prosocial behaviour	Important						
Psychosocial outcomes				Important			
Risk of comorbid conditions						Critical—HT, T2D, HIV	Critical—MS, SCI, ID
Sleep	Important	Important		Important			

*Outcomes are for subpopulation condition as listed: HT, T2D, MS, SCI, ID, PD, Stk, Sch, ADHD, MCD.

†Critical outcome: an outcome that is critical to decision-making. Important outcome: an outcome that is important, but not critical to decision-making.

ADHD, attention deficit hyperactivity disorder; CVD, cardiovascular disease; HT, hypertension; ID, intellectual disability; MCD, major clinical depression; MS, muscular sclerosis; PA, physical activity; PD, Parkinson’s disease; SB, sedentary behaviour; Sch, schizophrenia; SCI, spinal cord injury; Stk, in stroke survivors; T2D, type 2 diabetes.

Reviews that examined an association (based on levels above or below a threshold of physical activity or sedentary behaviour), and also reviews that explored the dose-response relationship between these behaviours and health-related outcomes were considered. In addition, six new umbrella reviews were commissioned to address health outcomes and populations not addressed by the above recent national physical activity guidelines; these umbrella reviews focused on the health impact of physical activity in people living with HIV/AIDS, osteoporosis and sarcopenia, the prevention of falls in older adults, the risk of adverse outcomes in adults and the health impacts of occupational physical activity.

The GDG reconvened in February 2020 to review the evidence and finalise a draft set of recommendations. They examined the quality of research contributing to each outcome identified in the PI/ECO questions and assessed the overall certainty of evidence (table 2) taking into consideration the risk of bias, inconsistency, imprecision, indirectness of the evidence and publication bias across each outcome, using the GRADE framework to rate the certainty of the evidence for each PE/ICO.^15^ Evidence profiles detailing this information for each PI/ECO are available.

The GDG considered the proposed wording of the recommendations and rated the strength of the recommendations as strong or conditional (table 2) based on the balance of benefits to harms, the certainty of evidence, sensitivity to values and preferences and the potential impact on gender, social and health equity, as well as acceptability, feasibility and resource implications. The assessment of the overall certainty of the evidence for each population group was based on an assessment across all evaluated outcomes (table 2). The GDG prioritised all-cause mortality and cardiovascular mortality as the most critical outcomes, followed by other clinical outcomes (eg, falls, depression, cognition, health-related quality of life, etc), then intermediate outcomes (eg, cardiometabolic markers, other metabolic markers) as well as physical activity risk and harms. Where there was a lack of subpopulation-specific evidence, the evidence for the general population was extrapolated but downgraded due to indirectness, when appropriate. The GDG came to consensus on each recommendation as well as the strength of recommendation ratings and voting was not required.

As required by the WHO process for guideline development, the draft guidelines were externally reviewed by seven independent reviewers, who provided feedback on the scientific evidence, its interpretation and the content of the guidelines. In addition, the draft guidelines and the evidence profiles were made available to the public and stakeholders, and feedback was sought through a global online consultation conducted between March and April 2020 and that received over 400 contributions. These inputs from scientists, practitioners and the general public were collated and used by the GDG to finalise the guidelines. These were approved by the WHO Guidelines Review Committee in August 2020.

## The 2020 guidelines on physical activity and sedentary behaviour

The final recommendations on physical activity and sedentary behaviour for each population group are summarised in table 4. For all populations, doing some physical activity is better than doing none. If individuals are not currently meeting these recommendations, doing some physical activity will bring benefits to health. Individuals should start with small amounts of physical activity and gradually increase frequency, intensity and duration over time. The GDG concluded that the benefits of doing physical activity and limiting sedentary behaviour outweighed the potential harms. Any potential harms may be managed by a gradual increase in the amount and intensity of physical activity.

**Table 4:** Summary of the WHO Guidelines on physcial activity and sedentary behaviour.

These public health guidelines are for all populations across the age groups from 5 years of age and above, irrespective of gender, cultural background or socioeconomic status and are relevant for people of all abilities. Those with chronic medical conditions and/or disability and pregnant and postpartum women should try to meet these recommendations where possible and as able.		
	Physical activity	Sedentary behaviour
**Children and adolescents** ***(aged 5–17 years), including those living with disability***	In children and adolescents, physical activity confers benefits for the following health outcomes: physical fitness (cardiorespiratory and muscular fitness), cardiometabolic health (blood pressure, dyslipidaemia, glucose and insulin resistance), bone health, cognitive outcomes (academic performance, executive function) and mental health (reduced symptoms of depression) and reduced adiposity. It is recommended that: Children and adolescents should do at least an average of 60 min/day of moderate-to-vigorous intensity, mostly aerobic, physical activity, across the week; Vigorous-intensity aerobic activities, as well as those that strengthen muscle and bone should be incorporated at least 3 days a week. *Strong recommendation*	In children and adolescents, higher amounts of sedentary behaviour are associated with detrimental effects on the following health outcomes: fitness and cardiometabolic health, adiposity, behavioural conduct/pro-social behaviour and sleep duration. It is recommended that: Children and adolescents should limit the amount of time spent being sedentary, particularly the amount of recreational screen time. *Strong recommendation*
**Adults** ***(aged 18–64 years) including those with chronic conditions and those living with disability***	In adults, physical activity confers benefits for the following health outcomes: all-cause mortality, cardiovascular disease mortality, incident hypertension, incident type 2 diabetes, incident site-specific cancers, mental health (reduced symptoms of anxiety and depression), cognitive health and sleep; measures of adiposity may also improve. It is recommended that: All adults should undertake regular physical activity; Adults should do at least 150–300 min of moderate-intensity aerobic physical activity, or at least 75–150 min of vigorous-intensity aerobic physical activity, or an equivalent combination of moderate-intensity and vigorous-intensity activity throughout the week for substantial health benefits; Adults should also do muscle-strengthening activities at moderate or greater intensity that involve all major muscle groups on 2 or more days a week, as these provide additional health benefits. *Strong recommendation* Adults may increase moderate-intensity aerobic physical activity to >300 min, or do >150 min of vigorous-intensity aerobic physical activity, or an equivalent combination of moderate-intensity and vigorous-intensity activity throughout the week for additional health benefits (when not contraindicated for those with chronic conditions). *Conditional recommendation*	In adults, higher amounts of sedentary behaviour are associated with detrimental effects on the following health outcomes: all-cause mortality, cardiovascular disease mortality and cancer mortality and incidence of cardiovascular disease, type 2 diabetes and cancer. It is recommended that: Adults should limit the amount of time spent being sedentary. Replacing sedentary time with physical activity of any intensity (including light intensity) provides health benefits; To help reduce the detrimental effects of high levels of sedentary behaviour on health, adults should aim to do more than the recommended levels of moderate-to-vigorous physical activity. *Strong recommendation*
**Older adults** ***(aged 65 years and older) including those with chronic conditions and those living with disability***	In older adults, physical activity also helps prevent falls and falls-related injuries and declines in bone health and functional ability. It is recommended that: As for adults, plus As part of their weekly physical activity, older adults should do varied multicomponent physical activity that emphasises functional balance and strength training at moderate or greater intensity on 3 or more days a week, to enhance functional capacity and to prevent falls. *Strong recommendation*	As for adults *Strong recommendation*
**Pregnant and postpartum women**	In women, physical activity during pregnancy and the postpartum period confers benefits for the following maternal and fetal health outcomes: reduced risk of pre-eclampsia, gestational hypertension, gestational diabetes, excessive gestational weight gain, delivery complications and postpartum depression and no increase in risk of stillbirth, newborn complications or adverse effects on birth weight. It is recommended that all pregnant and postpartum women without contraindication should: undertake regular physical activity throughout pregnancy and post partum; do at least 150 min of moderate-intensity aerobic physical activity throughout the week for substantial health benefits; incorporate a variety of aerobic and muscle-strengthening activities. Adding gentle stretching may also be beneficial. In addition: Women who, before pregnancy, habitually engaged in vigorous-intensity aerobic activity or who were physically active can continue these activities during pregnancy and the postpartum period. *Strong recommendation*	Pregnant and postpartum women should limit the amount of time spent being sedentary. Replacing sedentary time with physical activity of any intensity (including light intensity) provides health benefits. *Strong recommendation*
***Additional explanatory and practical notes***: *Some physical activity is better than none*. If not currently meeting these recommendations, doing some physical activity will bring benefits to health. Start with small amounts of physical activity and gradually increase frequency, intensity and duration over time. Pre-exercise medical clearance is generally unnecessary for individuals without contraindications prior to beginning light-intensity or moderate-intensity physical activity not exceeding the demands of brisk walking or everyday living. It is important to provide all children and adolescents with safe and equitable opportunities and encouragement to participate in physical activities that are appropriate for their age and ability, that are enjoyable, and that offer variety. Older adults should be as physically active as their functional ability allows and adjust their level of effort for physical activity relative to their level of fitness. When not able to meet the recommendations, adults with chronic conditions should aim to engage in physical activity according to their abilities. Adults with chronic conditions may wish to consult with a physical activity specialist or healthcare professional for advice on the types and amounts of activity appropriate for their individual needs, abilities, functional limitations/complications, medications and overall treatment plan. If pregnant and postpartum women are not currently meeting these recommendations, doing some physical activity will bring benefits to health. They should start with small amounts of physical activity and gradually increase frequency, intensity and duration over time. Pelvic floor muscle training may be performed on a daily basis to reduce the risk of urinary incontinence. Additional on safety considerations when undertaking physical activity for pregnant women are: Avoid physical activity during excessive heat, especially with high humidity; Stay hydrated by drinking water before, during and after physical activity; Avoid participating in activities which involve physical contact, pose a high risk of falling or might limit oxygenation (such as activities at high altitude, when not normally living at altitude); Avoid activities in supine position after the first trimester of pregnancy; Pregnant women considering athletic competition or exercising significantly above the recommended guidelines should seek supervision from a specialist healthcare provider; Pregnant women should be informed by their healthcare provider of the danger signs for when to stop, or limit physical activity and to consult a qualified healthcare provider immediately if they occur. Return to physical activity gradually after delivery and in consultation with a healthcare provider in the case of delivery by caesarean section. There are no major risks to people living with disability engaging in physical activity when it is appropriate to an individual’s current activity level, health status and physical function and the health benefits accrued outweigh the risks. People living with disability may need to consult a healthcare professional or other physical activity and disability specialist to help determine the type and amount of activity appropriate for them.		

Pre-exercise medical clearance is generally unnecessary. Individuals who are not currently regularly active and have no contraindications can be recommended to commence and gradually increase levels and intensity of physical activity without a medical clearance. An individual who is habitually engaging in moderate-intensity activity can gradually increase to vigorous intensity activity without needing to consult a healthcare provider. Those who develop new symptoms when increasing their levels of activity should consult a healthcare provider. These guidelines are for the general population and do not address the benefits and harms experienced by athletes undertaking the types and amounts of activity necessary to improve performance-related fitness for participation in competition.

The evidence supporting each of the updated or new recommendations is summarised for each group. Further details, including a more detailed narrative summary of evidence and the evidence profile tables summarising the evidence used for all recommendations, are available from WHO.^5^

### Recommendations for children and adolescents (5–17 years)

The evidence affirmed that physical activity in children and adolescents is associated with improved physical, mental and cognitive health outcomes. Many of the benefits of physical activity are observed with an average of 60 min of moderate-to-vigorous physical activity (MVPA) daily, although physical activity beyond 60 min of MVPA daily provide additional health benefits. There was insufficient evidence to determine whether specific health benefits vary by type or domain of physical activity. The evidence showed clearly that increased time in aerobic MVPA increases cardiorespiratory fitness and that increased muscle-strengthening activities increases muscular fitness, with some evidence showing incremental benefits of doing both. One notable update from the 2010 guidelines was evidence to support changing from ‘at least’ 60 min to ‘an average of’ 60 min of MVPA per day as this was deemed to more closely reflect the body of evidence and the way MVPA has been measured. The physical activity recommendation was rated as strong based on overall moderate certainty evidence. The evidence indicated that greater time spent in sedentary behaviour is related to adverse health outcomes. The association between sedentary behaviour and adverse health outcomes is generally stronger for television viewing or recreational screen time as the specific exposure variable than for total sedentary time in youth. There was, however, insufficient evidence to set a precise threshold (or ‘cut-off’) for the amount of sedentary or recreational screen time. The sedentary behaviour recommendation was rated as strong based on low certainty evidence.

### Recommendations for adults (18–64 years)

The evidence reaffirms that all adults should regularly undertake physical activity and that some physical activity is better than none. The adult guidelines include strong recommendations based on overall moderate-certainty evidence on weekly volumes of aerobic and muscle-strengthening physical activity. Many of the benefits of physical activity are observed within average weekly volumes of 150–300 min of moderate intensity or 75–150 min of vigorous intensity, or an equivalent combination of MVPA. The weekly range of recommended aerobic activity volume is a notable difference compared with the 2010 WHO recommendations that only specified *minimum* weekly thresholds. MVPA bouts of *any duration* now count towards these recommendations, reflecting new evidence to support the value of total physical activity volume, regardless of bout length.^16^ This recommendation differs from the requirement of bouts of at least 10 min in the previous WHO 2010 guidelines.

There is moderate-certainty evidence of a curvilinear dose-response association between physical activity volume and some health outcomes, such as all-cause and cardiovascular disease (CVD) mortality, and incident cancer and diabetes. Health benefits occur with levels of physical activity below the recommendations, supporting the statement that some physical activity is better than none. More physical activity is better, although the relative benefits tend to diminish at higher levels of physical activity. However, it is not possible to specify the physical activity levels where diminishing returns begin. For this reason, the new recommendation that aerobic physical activity volumes higher than 300 min of moderate-intensity activity per week, or 150 min of vigorous-intensity activity per week have additional health benefits, is rated as conditional. Beyond aerobic physical activity, additional health benefits will occur through participation in muscle-strengthening activities at moderate or greater intensity on 2 or more days a week, a strong recommendation supported by moderate-certainty evidence. There was no evidence to support a dose-response association with higher volumes of muscle-strengthening activities.

There was insufficient evidence to determine whether specific health benefits vary by type or domain of physical activity. Physical activity accrued at work, leisure, home or during transportation count towards the recommended amounts.

The reviewed evidence on sedentary behaviour and health outcomes in adults provided support that all adults should limit the amount of time spent sedentary. There was moderate-certainty evidence that the relationship of sedentary behaviour with all-cause and CVD mortality varies by amount of physical activity. For other outcomes, the evidence was insufficient. New evidence on the interdependent relationship between sedentary behaviour and physical activity underpinned the additional guidance that recommends increased levels of MVPA in the context of high levels of sedentary time. However, there was insufficient evidence to specify quantitative thresholds of sedentary behaviour, to determine whether specific health benefits vary by type or domain of sedentary behaviour or to determine the influence of frequency and duration of breaks in sedentary behaviour on health outcomes.

### Recommendations for older adults (65 years and above)

The evidence reviewed on physical activity and sedentary behaviour for adults also applied to older adults for the common set of critical health outcomes (table 3), because the majority of studies employed no upper age limit and therefore included adults over the age of 65 years. Additional health-related outcomes were reviewed because of their significant importance to older adults: 1) falls; 2) fall-related injuries; 3) physical function; 4) frailty and 5) osteoporosis.

New high-certainty evidence demonstrates an inverse dose-response relationship between volume of aerobic physical activity and risk of physical functional limitations in older adults. High-certainty evidence demonstrates that balance and functional exercises reduce the rate of falls and that engaging in a range of different types of physical activity can help to improve a wide range of elements of physical function. Moderate-certainty evidence indicates that the risk of fall-related injury may be reduced with multicomponent physical activity (combinations of balance, strength, endurance, gait and physical function training). As such it is recommended that as part of their weekly physical activity, older adults should do varied multicomponent physical activity at moderate or greater intensity on 3 or more days a week in order to enhance functional capacity and prevent falls. One notable update from the previous 2010 guidelines is that regular participation in this type of physical activity is recommended for *all* older adults rather than specifically those with poor mobility. Moderate-certainty evidence indicates that programmes involving multiple exercise types probably have significant effects on bone health and osteoporosis prevention. Because the evidence reviewed for sedentary behaviour in adults included those over the age of 65 years, the adult recommendations were deemed to also apply for this population group.

### Recommendations for pregnant and postpartum women

There is high-certainty evidence that physical activity during pregnancy is associated with reduced gestational weight gain and reduced risk of gestational diabetes mellitus in pregnant women with overweight or obesity. There is high-certainty to moderate-certainty evidence that the incidence of gestational hypertension is no different between pregnant women who exercise and those receiving standard antenatal care.

Among pregnant women with overweight or obesity, there is low-certainty to moderate-certainty evidence to suggest no increased risk of low birth weight, small for gestational age or large for gestational age babies between women who are physically active and those in standard antenatal care. There is moderate-certainty evidence of a small, but significant, reduced risk of preterm birth in mothers who engaged in vigorous physical activity. Similarly, among pregnant women with overweight or obesity there was no significant difference in the risk of preterm birth between those who were physically active and those in standard antenatal care. Available evidence from intervention trials combining both aerobic and muscle-strengthening physical activity support the recommendation for regular strength training to be included for pregnant and postpartum women.

No direct evidence was reviewed on sedentary behaviour for this subpopulation; however, the GDG reviewed the evidence for general populations and concluded it was applicable. Therefore, the sedentary behaviour recommendations for adults are extrapolated to pregnant and postpartum women and the certainty of the evidence downgraded for indirectness.

### Recommendations for people living with chronic conditions

Physical activity is considered safe for adults living with the selected chronic conditions without contraindications, and the benefits generally outweigh the risks. Evidence was reviewed for the following chronic conditions: cancer, hypertension, type 2 diabetes and HIV. Greater physical activity is related to improved health outcomes in people living with coronary heart disease. Among adults with type 2 diabetes, there is high-certainty evidence that physical activity is associated with decreased risk of CVD mortality and decreased levels of haemoglobin A1c, blood pressure, body mass index and lipids. Among adults with hypertension, there is high-certainty evidence that physical activity decreases risk of progression of cardiovascular disease and reduces blood pressure, while there is moderate-certainty evidence that physical activity reduces the risk of CVD mortality. High-certainty evidence shows that physical activity performed postcancer diagnosis is related to lower risks of mortality from all causes and mortality from cancer in female breast cancer survivors and colorectal cancer survivors.

Given that large numbers of people are currently living with HIV and that antiretroviral therapy has become effective and widely available, HIV is now considered a chronic condition. Thus, evidence on people living with HIV are included in these guidelines for the first time. There was moderate-certainty evidence that in people living with HIV, physical activity enhances health-related quality of life, maximal oxygen consumption, exercise tolerance, general health and physical functioning. There was moderate-certainty to high-certainty evidence that regular physical activity did not result in significant change in viral load, CD4+ count or disease progression, and as such, persons living with HIV are not adversely affected by physical activity.

There was moderate-certainty to high-certainty evidence that physical activity decreased symptoms of anxiety and depression. In addition, there was moderate-certainty to high-certainty evidence that physical activity was associated with a reduction in body fat percentage and an increase in lean body mass, but not waist circumference or body mass index.

Although there was no direct evidence on sedentary behaviour for these subpopulations, the GDG considered and concluded that the evidence for general populations was applicable. Therefore, the sedentary behaviour recommendations for adults were extrapolated to adults living with these chronic diseases and the certainty of the evidence downgraded for indirectness.

### Recommendations for people living with disability

Evidence was reviewed for the following health conditions: multiple sclerosis, spinal cord injury, intellectual disability, Parkinson’s disease, stroke, major clinical depression, schizophrenia and attention deficit hyperactivity disorder (ADHD). The following health outcomes were examined: comorbidity, physical functioning, cognition and quality of life, but not all outcomes were assessed for each condition. This evidence was considered together with the evidence for those without disability and the resulting recommendations were extrapolated to be applicable to people with disability in general.

Physical activity is considered safe and beneficial for people living with disability without contraindications, and there are no major risks when it is appropriate to an individual’s current activity level, health status and physical functioning level. Adults living with disability may need to consult a healthcare professional or other physical activity and disability specialist to help determine the type and amount of activity appropriate for them.

In people with spinal cord injury, low-certainty and moderate-certainty evidence suggests physical activity reduces shoulder pain and improves vascular function in paralysed limbs. Insufficient evidence was available to determine the relationship between physical activity and comorbid conditions in individuals with intellectual disability or multiple sclerosis. There is high-certainty evidence showing that physical activity can improve functioning in people with multiple sclerosis, spinal cord injury and a history of stroke. For people with intellectual disability or Parkinson’s disease, this evidence is of low certainty and high certainty, respectively. Limited evidence was available for the relationship between physical activity and quality of life in people with multiple sclerosis, spinal cord injury and intellectual disability. However, for people with schizophrenia and major clinical depression there was moderate-certainty evidence for beneficial effects on quality of life. Moderate-certainty evidence indicates that physical activity can have beneficial effects on cognition in people with multiple sclerosis, Parkinson’s disease, a history of stroke, ADHD and major clinical depression. For people with schizophrenia this evidence was of high certainty.

The evidence on the associations between sedentary behaviour and health outcomes in children, adolescents and adults living with disability was derived from literature reviewed for the general populations. The GDG concluded that these recommendations could be extrapolated to children, adolescents, adults and older adults living with disability, according to their specific ability, but downgraded the certainty of the evidence due to indirectness. The GDG agreed that benefits accrued from reducing sedentary time and gradually increasing physical activity where possible, depending on ability. In the case of those living with disability, especially wheelchair users or those with low mobility, it is important to note that it is possible to avoid sedentary behaviour while sitting or lying by doing light-intensity or high-intensity activities that do not involve the lower extremities.

## Discussion

The updated WHO 2020 *Guidelines on Physical Activity and Sedentary Behaviour* 5 provide clear, evidence-based, recommendations on how much physical activity provides health benefits for different population groups and on the potential risks of sedentary behaviours. These guidelines should be used to inform global, regional and national policy actions and investment, as well as to guide and strengthen national health behaviour surveillance systems that track progress towards national and global targets.^1^ The development of these new guidelines identified important areas requiring further research and thus also identified research priorities for the academic and research community.

### What remains the same?

The evidence confirms the value of participating in regular physical activity to achieve health benefits across all ages and abilities. Further it supports key messages that some physical activity is better than none and that more is better for optimal health outcomes. More specifically, the evidence reaffirms all adults should undertake regular physical activity and should aim to achieve at least 150 min of moderate-intensity, or 75 min of vigorous-intensity aerobic physical activity per week, or some equivalent combination of moderate-intensity and vigorous-intensity aerobic physical activity. Among children and adolescents, an average of 60 min/day of moderate-to-vigorous intensity physical activity across the week (most of which should be aerobic), leads to health benefits. Furthermore, the guidelines continue to reinforce the value of muscle-strengthening activity for all adults and children. The key physical activity-specific recommendations above remain largely unchanged from 2010 and are consistent with other recently developed physical activity guidelines from several countries.^13 17–22^

### What is new?

There are a few important differences in the new guidelines that should be highlighted for each age group. First, in adults, the previous stipulation that physical activity should be accumulated in at least 10 min bouts has been removed. This change reflects the accumulated evidence from cohort studies, which shows physical activity of any bout duration is associated with improved health outcomes, including all-cause mortality.^16 23^ Second, these updated guidelines for adults now specify a target range of 150–300 min of moderate-intensity and 75–150 min of vigorous-intensity physical activity, compared with the previous guidelines that focused on achieving at least 150 min of moderate-intensity or 75 min of vigorous-intensity activity per week. This change acknowledges that there is a range of physical activity which captures the maximal risk reductions for health outcomes associated with physical activity and going beyond this range does not appreciably further decrease the risk of major outcomes such as all-cause or CVD mortality. Third, with respect to older adults, the recommendation regarding multicomponent physical activity that emphasises functional balance and strength training to enhance functional capacity and prevent falls now applies to all older adults rather than specifically those with poor mobility. This change acknowledges the large volume of evidence demonstrating unequivocal beneficial effects of this physical activity type on the functional capacity and risk of falls in older people with a range of functional abilities.

There was also one key change in the new recommendations for children and adolescents. Specifically, the updated recommendation is now to do at least an ‘average of 60 min/day’ of MVPA rather than the previously stated ‘*accumulate* 60 min of physical activity daily’. Although this might appear to be a subtle difference, the change better reflects the scientific evidence as most studies reported associations between an average daily value of physical activity rather than an accumulation of 60 min on each and every day of the week.

The new recommendation that sedentary behaviour should be limited across all groups is an important addition to these new global guidelines since 2010 and is in line with other recent country-level guidelines^19 20^ that generally support the notion of moving more and reducing sedentary time (‘sitting less’ for ambulatory people). Although specifying a quantitative threshold on the amount of sedentary behaviour was strongly considered, there was insufficient evidence. Furthermore, variations in how sedentary time was measured, and that a threshold would likely vary by health outcome, by level of physical activity and by population subgroups, made quantifying upper time limits difficult to ascertain.

The recommendation to limit sedentary behaviour was qualified with an acknowledgement that replacing sedentary time with any intensity of physical activity (including light intensity) has health benefits. This recommendation was based first on the juxtaposed evidence of lower levels of time spent in sedentary behaviours being beneficial for health even among those with modest levels of MVPA, and on the emerging, largely cross-sectional evidence, from ‘replacement’ studies (ie, isotemporal substitution) demonstrating these effects more directly. Second, there was evidence of effect modification between sedentary time and MVPA, which supported the development of a second recommendation emphasising the benefits of undertaking more than the recommended levels of MVPA to help reduce the detrimental effects of high levels of sedentary behaviour. The important practical application of this recommendation is to encourage the promotion of multiple approaches to limiting the negative health outcomes associated with high levels of sedentary time. This includes recommending individuals reduce their time spent in sedentary behaviours or increase their MVPA to help offset the negative impact, or some combination of both strategies. Given that time spent in sedentary behaviours at work, for transport or for recreation appears to be overtaking time spent in more healthy physical activity behaviours during waking hours, the GDG deemed it important to attend to both physical activity and sedentary time and, therefore, to recommend a ‘balance’ of these behaviours for better health. Although the evidence on health benefits of ‘breaking up’ sedentary time and types of sedentary behaviours was reviewed, the GDG considered that there was insufficient evidence to provide specific quantified recommendations.

An additional issue related to the sedentary recommendations was the decision to opt specifically for the use of the term ‘sedentary behaviour’ instead of ’sitting’, which has been commonly used in several national guidelines. This wording choice was deliberate and made to reflect the overarching agenda of these new guidelines to be inclusive of people living with disability and therefore to emphasise options for reducing sedentary behaviour among wheelchair users and those with low mobility, where prolonged sitting may be unavoidable. For such people, sedentary time can be minimised through physical activity while remaining seated.

### Guidelines for special groups—key considerations

The development of recommendations on physical activity and sedentary behaviours specifically for key populations, namely, people living with disability and chronic conditions, as well as for pregnant and postpartum women, addressed important gaps in global health policy. These new recommendations affirm that physical activity is feasible for these groups; and provides for the first-time global science-based recommendations to inform the development of population-based initiatives to improve health outcomes for these population groups. In particular, these recommendations for people living with chronic disease and disability should stimulate increased attention in policy, surveillance, investment and research aligned to the agenda of inclusion as called for in the sustainable development goals^24^ as well as in the convention on the rights of people living with disability.^25^

In undertaking this new work there were however several limitations. Specifically, the association between physical activity and health outcomes was only reviewed for selected chronic conditions and disabilities and there was limited evidence to inform on the optimal type, frequency or duration of activity by health condition. Due to the lack of direct evidence, considerable extrapolation was needed to develop the recommendations on physical activity and the recommendations on sedentary behaviour relied entirely on the evidence from the general population. Nonetheless, the GDG concluded the strong recommendations for this population reflect the balance between desirable and undesirable consequences and send an important message to support the inclusion of people living with disability in physical activity population health initiatives.

### Using these guidelines

Developing global guidelines is not an end in itself. The 2020 WHO guidelines provide a set of evidence-based physical activity and sedentary behaviour recommendations that national governments can adopt, thus removing the need for countries to use limited resources to undertake their own scientific reviews and expert consensus process. This issue is particularly important for LMICs where resources may be limited. During adoption of these global guidelines, national policymakers are encouraged to consider their national context and factors such as culture, ethnic diversity, existing social norms and the current provision for physical activity promotion within healthcare services as well as primary prevention, to inform adaptations and dissemination of the guidelines.

The existence of guidelines, in isolation, is unlikely to lead to increases in population levels of physical activity. It is critical that they are supported by coordinated dissemination to key audiences and a sustained national public education communication strategy. Furthermore, communication activities must be combined with implementation of setting specific policy actions to support behaviour change. How to optimise the impact of physical activity guidelines through effective communication strategies is explored in a separate paper in this issue.^26^

In 2018, the new GAPPA 2018–2030 set a target to reduce physical inactivity by 15% by 2030 and outlined 20 recommended policy actions and interventions.^1^ These included recommending all countries combine sustained national public education and awareness campaigns with the integration of physical activity counselling programmes into primary and secondary healthcare. Other recommendations included the creation of appropriate and supportive environments for physical activity for all population groups and increasing opportunities for physical activity in schools, workplaces, cities and communities and as a form of safe and sustainable transport.

These 2020 global guidelines provide focus to the overall goal of national policy and support expanding the scope of actions to include additional groups, such as people living with disability, chronic conditions and women who are pregnant or post partum. National policy will need to offer a route to the development of appropriate programme delivery and practice that recognises community needs and the diversity of groups and contexts and seeks to reduce existing disparities in access to and engagement in physical activity. The inclusion of global recommendations on muscle strengthening activities is not new in these updated guidelines, but the GDG implicitly recognised their increasing importance due to an expanding evidence base. Promotion of muscle strengthening and falls prevention activities have been largely forgotten or ignored in the past^27 28^ and in most countries a much greater focus on this is now required for policy and practice.

### Implications of these guidelines for health surveillance

These updated guidelines have several implications for future population monitoring and research. First, currently used surveillance instruments and/or protocols will need adaptation to align with the key changes made in these updated guidelines. Second, national population surveillance systems will need to be extended to include and track trends in key populations such as children aged 5–10 years, pregnant and postpartum women, older adults and persons living with disability or chronic conditions. Third, monitoring systems should be strengthened to track trends in muscle-strengthening exercises, which are of increasing importance with an ageing demographic in many countries. Fourth, as many countries are reliant on self-reported methods, which have well-established limitations,^29^ there is a need to accelerate advancements in sensor technology to ensure it provides a practical and affordable approach to assessing physical activity and sedentary behaviours. The potential of the new guidelines for advancing surveillance as well as the need for development of device-based approaches to inform a new generation of guidelines is discussed by Troiano *et al*.^30^

### Key knowledge gaps

Despite the large quantity of data relating physical activity, and increasingly sedentary behaviours, to health outcomes across the life-span, the GDG discussions revealed important evidence gaps, which should be prioritised to inform future guidelines. The most common need cited is more research on the dose-response relationship between volume and/or intensity of physical activity and health outcomes. Such information is key to establishing minimal effective doses and maximum safety thresholds of physical activity for different population subgroups. There also remains limited evidence from LMICs and economically disadvantaged or underserved communities and many studies are not designed or powered to test for effect modification by various sociodemographic factors. Such information is important for making more specific public health recommendations and reducing health disparities in more vulnerable sectors of the population. Further details on the research gaps arising from these new guidelines can also be found elsewhere.^31^

## Conclusions

These new, updated WHO guidelines on physical activity and sedentary behaviour, together with the WHO guidelines for under 5 years of age,^6^ provide recommendations on physical activity and sedentary behaviour for individuals across the whole age spectrum and address a long-lasting gap with the inclusion of key populations for the first time. Collectively, the recommendations affirm the importance of regular aerobic and muscle-strengthening physical activity and reduction in sedentary behaviours. Benefits accrue from doing any amount of physical activity and this applies to people of all ages and abilities. There are significant health gains and cost savings to health systems if countries adopt these guidelines and direct efforts and resources to implementation of programmes and policy to enable achievement of the 2030 GAPPA target set out in the global action plan on physical activity.^1^ Benefits extend also beyond the health sector as mounting evidence across diverse fields shows the interconnected social, economic and environmental impacts of more physically active populations. Now, it is time to work to ensure and support the adoption and implementation of these new global guidelines for a healthier, more active future worldwide.

**Key messages**

- These new 2020 WHO *Guidelines on Physical Activity and Sedentary Behaviour* provide evidence-based public health recommendations concerning the amount and types of physical activity that offer significant health benefits and mitigate health risks.
- They update and replace the previous 2010 WHO recommendations on physical activity.
- The guidelines address children over the age of 5 years, adults, older adults and, for the first time, include specific recommendations for pregnant and postpartum women and people living with chronic conditions or disability.
- For all populations, the benefits of doing physical activity and limiting sedentary behaviour outweighed the potential harms.
- Risks can be managed by gradual increase in the amount and intensity of physical activity.
- Some physical activity is better than none for those not currently meeting these recommendations, individuals should start with small amounts of physical activity and gradually increase frequency, intensity and duration over time.
- Countries are encouraged to adopt and disseminate these new global guidelines to key audiences, and use them as the basis for sustained national public education communication campaigns responding to their national context and factors such as culture, ethnic diversity and social norms.
- These new guidelines should inform national policy and actions to promote physical activity and reduce sedentary behaviours as well as to align national health behaviour surveillance systems that track progress towards national and global targets.
- Important evidence gaps remain and more research is needed on the dose-response relationship between volume and/or intensity of physical activity and health outcomes, particularly in people living with disability, and further evidence from low-income and middle-income, disadvantaged or underserved communities.
